# Association between cystatin C gene polymorphism and the prevalence of white matter lesion in elderly healthy subjects

**DOI:** 10.1038/s41598-020-61383-7

**Published:** 2020-03-13

**Authors:** Kyohei Maniwa, Shozo Yano, Abdullah Md. Sheikh, Keiichi Onoda, Shingo Mitaki, Minoru Isomura, Seiji Mishima, Shuhei Yamaguchi, Toru Nabika, Atsushi Nagai

**Affiliations:** 1 Central Clinical Laboratory Division, Shimane University Hospital, Izumo, Japan; 2 Department of Laboratory Medicine, Shimane University Faculty of Medicine, Izumo, Japan; 3 Department of Neurology, Shimane University Faculty of Medicine, Izumo, Japan; 4 Shimane University Faculty of Human Sciences, Matsue, Japan; 5 Shimane Prefecture Hospital Bureau, Izumo, Japan; 6 Department of Functional Pathology, Shimane University Faculty of Medicine, Izumo, Japan

**Keywords:** Genetics, Neurology, Risk factors

## Abstract

Cystatin C (CST3) is a cysteine protease inhibitor abundant in the central nervous system, and demonstrated to have roles in several pathophysiological processes including vascular remodeling and inflammation. Previously, we showed a relation of *CST3* gene polymorphisms with deep and subcortical white matter hyperintensity (DSWMH) in a small case-control study. In this study, we aimed to investigate the relation in a larger cross-sectional study. Participants of a brain health examination program were recruited (n = 1795) in the study, who underwent routine blood tests and cognitive function tests. Cerebral white matter changes were analyzed by MRI. Additionally, 7 single nucleotide polymorphisms (SNPs) (−82G/C, −78T/G, −5G/A, +4A/C, +87C/T, +148G/A and +213G/A) in the promoter and coding regions of *CST3* gene were examined. Among them, carriers of the minor allele haplotype −82C/+4C/+148A were significantly associated with decreased CST3 concentration in the plasma. Unadjusted analysis did not show significant relation between carriers of the minor allele haplotype and periventricular hyperintensity (PVH), but DSWMH was marginally (*p* < 0.054) increased in this group. After adjusting the effects of other variables like age and kidney function, logistic regression analysis revealed that carriers of the minor allele haplotype were at a significantly increased risk of developing both PVH and DSWMH. Thus, our results suggest that carriers of the minor allele haplotype −82C/+4C/+148A of *CST3* gene could be at an increased risk to develop cerebral white matter disturbance.

## Introduction

Cystatin C (CST3) is a 13-kDa protein consisting of 120 amino acids, encoded by a 7.3-kb gene located in chromosome 20^1^. It is expressed in all types of cells^2^, where it is located in the lysosome, Golgi apparatus and endoplasmic reticulum^3–7^. It is also a secreted protein and found in all types of body fluid^8^. Particularly, CST3 concentration is high in cerebrospinal fluid (CSF), signifying its high expression and important function in central nervous system (CNS)^8,9^. It can inhibit the activity of cysteine proteases including cathepsin B, L and H, and is considered to be the main endogenous inhibitor of those enzymes^8^. Since these proteases have important roles in various pathophysiological processes including inflammation and immune regulation, vascular remodeling and cell migration, CST3 could affect those processes by modulating their activities^10–14^. Indeed, it is demonstrated that by modulating the activities of cysteine proteases, CST3 is shown to play an important role in the pathology of several disease processes including atherosclerosis, tumor metastasis, chronic kidney diseases and inflammatory conditions. For example, in cerebral aneurism, the activity of proteases is increased due to decreased CST3 levels, which cause excessive extracellular matrix degradation^10^. Also, abdominal aortic diameter is inversely correlated with serum CST3 levels^15^. On the other hand, increased serum CST3 concentration positively correlates with the plaque area and plaque burden in patients with unstable angina^16^. These findings suggest that a precise balance of proteases and CST3 is important for the vascular physiological processes. In addition to cardiovascular and peripheral vascular disease, CST3 can affect conditions related to CNS and cerebral vessel. In the processes of neuroinflammation, neurodegeneration and vascular remodeling, CST3 is demonstrated to be important^10,13,17^. In several disease conditions including amyotrophic lateral sclerosis, neuroinflammatory diseases and leptomeningeal metastasis, its concentration in CSF is altered^12,18,19^. Regarding cerebrovascular pathology, CST3 was found to be deposited in the vessel wall in hereditary cerebral amyloid angiopathy (CAA). In hereditary CAA, a point mutation in the gene causes substitution of leucine to glutamine at position 68 (L68Q) in the amino acid sequence of protein^20^. In contrast, in non-hereditary CAA, especially in Alzheimer’s disease (AD), CST3 is usually co-deposited with amyloid β (Aβ) peptide indicating its important role in the disease pathology^21^.

In addition to L68Q, several other nucleotide changes in the promoter and coding sequence of *CST3* gene have been reported. Among single nucleotide polymorphisms (SNP) in *CST3* gene, −82G/C (rs5030707) located in the 5′-promoter region has been reported to affect promoter activity^22^. Another SNP + 148G/A (rs1064039) located in the coding region causes the changes in CST3 secretion^23^. Furthermore, a haplotype of 3 SNPs containing −82G/C, +4A/C (rs4994881) and +148G/A has been reported to be associated with CST3 levels in serum and CSF^22^. Since CSF profile broadly represents the pathophysiology of CNS, useful information might be obtained by investigating the effects of gene polymorphism on CSF CST3 concentration in relation to CNS small vessel diseases. Our previous case-control study demonstrated that the haplotype of 3 SNPs in *CST3* gene (−82C/+4C/+148A) is related to lower plasma CST3 concentration and risk of severe cerebral white matter lesion^24^. Since the study was done in a small and targeted population, a large-scale study is warranted to further clarify the association of *CST3* polymorphism with white matter disease occurrence and cognitive function in general population. Therefore, the aim of this study is to examine the relation of white matter diseases with *CST3* SNPs in Japanese healthy population, and confirm whether plasma CST3 levels are affected by the SNPs. Such information would be valuable to understand the role of protease systems in pathophysiology of cerebral white matter diseases.

## Results

### Demographic data of the study population

Personal and health related history and the clinical characteristics of the study population (n = 1795) are shown in Table 1. Among the study population, 599 subjects were identified as PVH positive (grade 1–3), and 828 subjects as DWMH positive (grade 1–3). The average plasma concentration of CST3 of the study population was 0.85 ± 0.16 mg/L.

**Table 1:** Clinical Characteristics of the study population.

	Value (n = 1795)
Age (years)	60.1 ± 8.9
Male/Famale	1002/793
History of hypertension (%)	35.0
History of diabetes (%)	16.3
History of hyperlipidemia (%)	62.3
Current smokers (%)	14.8
Current alcohol drinkers (%)	42.8
School education (years)	12.9 ± 2.5
Body mass index (kg/m^2^)	23.2 ± 3.09
Systolic BP (mm Hg)	128.3 ± 17.1
Diastolic BP (mm Hg)	73.3 ± 11.2
Fasting plasma glucose (mg/dL)	103.4 ± 23.1
HbA1c (JDS) (%)	5.5 ± 0.8
eGFR (mL/min/1.73 m^2^)	77.2 ± 13.6
PVH	
Positive (grade 1–3)	599
Negative (grade 0)	1196
grade0	1196
grade1	451
grade2	124
grade3	24
DSWMH	
Positive (grade 1–3)	828
Negative (grade 0)	967
grade0	967
grade1	528
grade2	271
grade3	29
Plasma CST3 (mg/L)	0.85 ± 0.16

### Genotype frequency and allele frequency

Genotyping for −82 G/C, −5 G/A, +4A/C, +87C/T, +148G/A and +213G/A in *CST3* gene was done in 1795 subjects. Table 2 shows the genotype frequencies and allele frequencies of each SNP. No polymorphism was found at positions -5 G/A (rs113065546), +87C/T (rs1055084) and +213G/A (rs2010109955) in our study subjects, and was not considered for further analysis. Moreover, +87C/T and +213G/A polymorphisms do not change the amino acid sequence, and probably do not have functional importance. Remaining three polymorphisms at position −82, +4 and +148 were in concordance with Hardy-Weinberg equilibrium.

**Table 2:** Genotype frequency and allele frequency.

n = 1795	Genotype frequency, n (%)			Allele frequency	
	GG	GC	CC	G	C
−82	1362(75.9)	402(22.4)	31(1.7)	0.87	0.13
	AA	AC	CC	A	C
+4	1358(75.7)	406(22.6)	31(1.7)	0.87	0.13
	GG	GA	AA	G	A
+148	1359(75.7)	403(22.5)	33(1.8)	0.87	0.13
	GG				
−5 & +213	1795				
	CC				
+87	1795				

### Relation of genotype frequency and clinical characteristics

The relation of polymorphic genotype frequencies and clinical characteristics is shown in Table 3. Haplotype analysis at position −82, +4 and +148 revealed a major allele haplotype of G/A/G (allele A), and a minor allele haplotype of C/C/A (allele B). Since the number of subjects with homozygous BB (homozygous minor allele haplotype) was very small, subsequent analysis was conducted to compare between homozygous AA and carriers of the B allele (homozygous BB and heterozygous AB). The data of 20 subjects were excluded from the analysis because they did not fall into the category of homozygous AA or carriers of the B allele. The concentration of plasma CST3 in carriers of the B allele was significantly lower compared to that of homozygous AA (*p* < 0.001). On the other hand, the prevalence of DSWMH was marginally increased in carriers of the B allele (*p* = 0.054). When other parameters were considered, no significant differences were found between the groups.

**Table 3:** Relationship of *CST3* gene polymorphisms and clinical characteristics.

Carrier allele	AA	AB + BB	
SNPs (−82/+4/+148)	(GG/AA/GG)	(GC/AC/GA) and (CC/CC/AA)	
n	1352	423	p
Plasma CST3 (mg/L)	0.86 ± 0.16	0.83 ± 0.15	<0.001
Age, years	60.3 ± 8.84	59.8 ± 9.16	0.303
Male (%)	56.0	55.8	0.943
History of hypertension (%)	34.7	36.4	0.537
History of diabetes (%)	16.3	16.5	0.893
History of hyperlipidemia (%)	61.8	63.6	0.515
Current smokers (%)	15.1	13.7	0.486
Current alcohol drinkers (%)	43.2	42.1	0.686
School education (years)	12.9 ± 2.52	13.1 ± 2.52	0.085
Body mass index (kg/m^2^)	23.2 ± 3.01	23.1 ± 2.79	0.572
Systolic BP (mm Hg)	128.3 ± 17.2	128.1 ± 16.9	0.998
Diastolic BP (mm Hg)	73.3 ± 11.2	73.1 ± 11.1	0.926
Fasting plasma glucose (mg/dL)	103.2 ± 29.5	104.2 ± 28.1	0.621
HbA1c (JDS) (%)	5.46 ± 0.73	5.46 ± 0.85	0.249
eGFR (mL/min/1.73 m^2^)	77.1 ± 13.4	77.8 ± 14.3	0.272
PVH	0.42 ± 0.68	0.48 ± 0.70	0.096
DSWMH	0.63 ± 0.79	0.71 ± 0.81	0.054
Okabe’s Intelligence Scale	44.8 ± 7.15	44.8 ± 7.12	0.984
Kohs Block Design Test	101.8 ± 18.4	102.6 ± 18.3	0.460
Self-rating Depression Scale (SDS)	34.6 ± 7.53	34.5 ± 7.84	0.707
Apathy Scale	11.1 ± 5.75	11.2 ± 5.50	0.755
Frontal Assessment Battery (FAB)	16.0 ± 1.45	16.0 ± 1.45	0.600

*PVH and DSWMH are expressed by the grading scale.

### Relationship between PVH and CST3 gene polymorphism

In Table 4, the relation of PVH with the clinical characteristics is shown. The analysis demonstrated that several parameters including age, history of hypertension, history of diabetes, duration of school education, systolic BP and fasting blood glucose were significantly associated with PVH (*p* < 0.001). PVH also significantly associated with eGFR (*p* < 0.003). Logistic regression analysis showed that carriers of the B allele were 1.285 times more likely to have PVH (*p* = 0.044), while gender, history of hyperlipidemia, smoking history, BMI and eGFR did not increase the risk (Table 5).

**Table 4:** Clinical characteristics of PVH negative or positive person.

PVH	Negative	Positive	p
n	1196	599	
Plasma CST3 (mg/L)	0.85 ± 0.15	0.87 ± 0.18	0.09
Age, years	58.2 ± 8.82	63.9 ± 7.81	<0.001
Male (%)	56.4	54.8	0.521
History of hypertension (%)	29.7	45.7	<0.001
History of diabetes (%)	14	20.7	<0.001
History of hyperlipidemia (%)	61.9	63.3	0.564
Current smokers (%)	15.9	12.5	0.058
Current alcohol drinkers (%)	44.4	39.7	0.06
School education (years)	13.2 ± 2.45	12.4 ± 2.58	<0.001
Body mass index (kg/m^2^)	23.3 ± 3.00	23.1 ± 2.85	0.848
Systolic BP (mm Hg)	127.1 ± 16.8	130.5 ± 17.4	<0.001
Diastolic BP (mm Hg)	73.4 ± 11.2	73 ± 11.2	0.469
Fasting plasma glucose (mg/dL)	101.7 ± 20.2	106.6 ± 27.7	<0.001
HbA1c (JDS) (%)	5.43 ± 0.69	5.52 ± 0.87	0.138
eGFR (mL/min/1.73 m^2^)	77.8 ± 13.0	76.1 ± 14.7	0.003
Carrier of B allele (%)	22.4	25.9	0.113

**Table 5:** Logistic regression analysis of PVH.

Independent variables	OR	95% CI		p
		Lower	Upper	
Age	1.077	1.062	1.093	<0.001
Sex	0.847	0.646	1.112	0.232
History of hypertension	1.641	1.311	2.054	<0.001
History of diabetes	1.329	1.007	1.755	0.045
History of hyperlipidemia	0.985	0.787	1.232	0.894
Current smokers	1.041	0.749	1.446	0.811
Current alcohol drinkers	0.753	0.580	0.976	0.032
School education	0.911	0.872	0.952	<0.001
Body mass index	0.985	0.949	1.023	0.443
eGFR	1.001	0.993	1.009	0.839
Carrier of B allele	1.285	1.007	1.642	0.044

### Relationship between DSWMH and *CST3* gene polymorphism

In Table 6, the relation of DSWMH with the clinical characteristics is shown. The analysis revealed that the parameters including age, history of hypertension, history of diabetes, current smokers, duration of school education, systolic BP, fasting blood glucose and eGFR were significantly associated with DSWMH. Importantly, plasma CST3 level was significantly higher in the subject group positive for DSWMH (*p* < 0.001). Logistic regression analysis showed that carriers of the B allele were 1.301 times more likely to have DSWMH, while gender, history of diabetes, history of hyperlipidemia, smoking history, drinking history, school education, BMI and eGFR did not increase the risk (Table 7).

**Table 6:** Clinical characteristics of DSWMH negative or positive person.

DSWMH	Negative	Positive	p
n	967	828	
Plasma CST3 (mg/L)	0.83 ± 0.14	0.88 ± 0.17	<0.001
Age, years	57.3 ± 8.56	63.4 ± 8.13	<0.001
Male (%)	56.5	55.1	0.554
History of hypertension (%)	27.9	43.4	<0.001
History of diabetes (%)	13.5	19.4	0.001
History of hyperlipidemia (%)	63	61.6	0.546
Current smokers (%)	17	12.2	0.005
Current alcohol drinkers (%)	42.5	43.2	0.754
School education (years)	13.1 ± 2.49	12.7 ± 2.55	0.001
Body mass index (kg/m^2^)	23.3 ± 2.94	23.2 ± 2.96	0.573
Systolic BP (mm Hg)	126 ± 16.6	130.8 ± 17.2	<0.001
Diastolic BP (mm Hg)	72.9 ± 11.3	73.7 ± 11.0	0.089
Fasting plasma glucose (mg/dL)	102.2 ± 21.8	104.7 ± 24.4	0.003
HbA1c (JDS) (%)	5.43 ± 0.70	5.5 ± 0.81	0.246
eGFR (mL/min/1.73 m^2^)	78.2 ± 12.5	76.1 ± 14.7	<0.001
Carrier of B allele (%)	21.8	25.6	0.076

**Table 7:** Logistic regression analysis of DSWMH.

Independent variables	OR	95% CI		p
		Lower	Upper	
Age	1.090	1.075	1.106	<0.001
Sex	1.029	0.793	1.334	0.832
History of hypertension	1.575	1.266	1.958	<0.001
History of diabetes	1.255	0.953	1.654	0.106
History of hyperlipidemia	0.851	0.688	1.053	0.139
Current smokers	0.906	0.667	1.231	0.527
Current alcohol drinkers	1.057	0.826	1.353	0.660
School education	0.987	0.947	1.029	0.538
Body mass index	0.988	0.953	1.024	0.513
eGFR	1.000	0.992	1.008	0.975
Carrier of B allele	1.301	1.027	1.647	0.029

## Discussion

To determine the effect of the genetic variations in *CST3* gene on cerebral white matter changes, 7 polymorphisms (−82G/C, −78T/G, −5G/A, +4A/C, +87C/T, +148G/A and +213G/A) in *CST3* gene have been analyzed, and checked their relation with laboratory data, cognitive impairment and MRI findings in healthy Japanese subjects. The analysis revealed that in the study population, there was no polymorphism at −5, +87 and +213 positions in the gene. Since −78T/G and −82G/C was haplotype, 3 polymorphisms at −82G/C, +4A/C and +148G/A were chosen for further analysis. Our analysis demonstrated that the polymorphism at these three positions was the haplotype of *CST3* gene that affected the plasma concentration and brain white matter lesions.

Several studies have demonstrated that the polymorphism in *CST3* gene can affect its production and secretion from the cells, and its concentration in serum and cerebrospinal fluid^23–27^. Interestingly, a study found that the minor allele carriers at −82, −78, −5 and +148 positions had lower plasma CST3 concentration^19^. Since −82, −78 and −5 are in the gene regulatory region, it is conceivable that the decreased level of CST3 might be caused by the suppression of transcriptional activity. Consequently, it was suggested that the mutation at −82 position caused decreases CST3 promoter activity^22^. Mutation at +148 position in CST3 mRNA alters the amino acid sequence near the end of the signal peptide. Since that position is important for protein maturation and subsequent secretion, we reasoned that polymorphism at +148 could alter the secretion of the protein. Indeed, we have shown in our previous study that +148A allele is critical for CST3 secretion^24^. Hence, the decreased plasma CST3 levels in minor allele carriers might have resulted from decreased promoter activity as well as secretion. Due to reduced secretion caused by +148A, intracellular CST3 level was increased^24^, which could alter intracellular protease activity. Such alteration of the balance of intracellular protease activity might induce degenerative changes that are manifested as cerebral white matter hyperintensities.

In our study, the population with carriers of the B allele had lower CST3 level. However, the subject group with white matter disturbance demonstrated higher plasma CST3 levels. This subject group is aged, and eGFR is lower than that of the subject group with no white matter disturbance. Since plasma CST3 is exclusively cleared by the kidney, higher CST3 level in the subject group with cerebral white matter disturbance could be explained by decreased renal function. Interestingly, after adjustment, logistic regression revealed an increased risk of PVH and DHWMS in B allele. As we discussed above, B allele can affect the production and secretion of CST3. Then its level in cerebrovascular tissue could decrease despite high plasma concentration. Taken together, these findings suggest a relation of cerebral small vessel disease and the alteration of CST3 expression due to polymorphism that is independent of kidney-mediated clearance.

Previous studies have demonstrated that both increased and decreased CST3 levels seem to increase cerebral small vessel diseases pathology^24,28,29^. Such discrepancy has also been observed in atherosclerotic conditions. Several studies showed that higher CST3 levels are associated with a higher plaque score in atherosclerotic lesions, increased risk of subsequent cardiovascular events, and independently predictive of incident peripheral arterial disease^16,30,31^. In another study, the subject group with minor allele haplotype of *CST3* gene has lower CST3 serum levels, and a higher average number of coronary artery stenosis^22^. Although the mechanism of such discrepancy in atherosclerotic condition is still unknown, the probable cause might be the imbalance of the activity of cysteine protease cathepsins and their endogenous inhibitor CST3 in atherosclerotic lesions. Similar function of CST3 could be important in cerebrovascular disease pathology. Nevertheless, we had a concern that *CST3* polymorphism affects atherosclerotic lesions, which might change cerebral small vessel diseases pathology indirectly. Our additional analysis revealed that there was no significant association of carriers of the B allele with general atherosclerotic factors such as intima-media thickness of cervical artery (data not shown). Hence, white matter disturbance in the subject group might not be caused by atherosclerosis, rather directly affected by CST3-mediated alteration of protease activities.

In our previous study^24^, we suspected a relationship between low CST3 plasma levels and cerebral white matter lesions due to a significant association of carrier of the B haplotype and serum CST3 concentration with the condition. CSF CST3 is considered predominantly as a brain-derived protein^32–35^, where its concentration is 5.5 times higher than serum. Several cell types of CNS produce CST3 including astrocytes, leptomeningeal cells and cells of choroid plexus. Hence, CNS microenvironment might influence CST3 level in CSF independent of the serum levels. In the conditions like inflammatory CNS diseases^12^ and leptomeningeal carcinomatosis^18^, the protease production is increased leading to an imbalance of the activity and altered tissue remodeling. On the other hand, in hereditary CAA, CST3 levels are decreased in CSF. Here, its truncated form is aggregated and deposited in cerebral vessels, where it co-localized with Aβ peptide and predominantly deposited in the adventitial layer of vessel wall. Hence, the interaction of these 2 molecules occurs in the brain or in CSF^36^, not in arterial intraluminal space. Such aggregation and deposition alter protease inhibitory activity of CST3. Because CST3 levels are thought to be balanced with cysteine proteases activities in healthy conditions, a disturbance of such balance could be caused by its decreased production, aggregation and deposition. Similar imbalance of protease activity possibly occurs in carriers of the B allele where the production and the concentration of CST3 in CSF decreased^22,37^. Consequently, extracellular matrix remodeling and elastin degradation in arterial wall^15,38^, brain arteriole remodeling and inflammation caused by excessive protease activity might lead to white matter hypoperfusion and higher incidence of brain white matter lesion.

To understand the genetic influence in the pathology of cerebral small vessel diseases, genome wide association studies (GWAS) have been conducted to identify the contributing loci in the chromosome^39,40^. Several loci for the disease were found on chr17q25, chr10q24 and chr2p21, but none of them harbor *CST3* gene. However, most of the study populations were from European descent. Since GWAS result can be influenced by ethnicity^41^, it is possible that additional loci can be found in cerebral small vessel disease GWAS study of Japanese population that harbor *CST3* gene.

Progression of white matter lesion such as periventricular white matter hyperintensity is a predictor of cognitive impairment risk in healthy elderly individuals^42^. Although initiation of white matter damage is more likely to be induced in carriers of the B allele, any of the cognitive test performances were not different between the groups. The reason might be the tests parameters were not affected at the early stage of white matter damage. It would be interesting to know whether the relation of white matter damage and decline of cognitive performance is evident in carriers of the B allele in next follow-up study. There are also other limitations in this study. Our study recognized that although carriers of the B allele have lower level of plasma CST3, it might not be the same in its CSF level. In future study, we need to confirm the effect of polymorphism on CSF CST3 levels as well. Another important aspect can be explored is *CST3* gene relationship with AD and APOE genotype. Moreover, other class II cystatins could be important for cerebral small vessel disease pathology. Particularly cystatin F is increased in the CSF of AD patients^43^, indicating its relation with neurodegenerative conditions. It will be interesting to explore its relation with cerebral small vessel diseases.

In conclusion, this study has shown that the carriers of the B allele (−82C/+4C/+148A) have lower levels of serum CST3. The population did present MRI evidence of cerebral small vessel disease without the decline of cognitive performance, indicating the involvement of CST3 from the early stage of the disease. Such findings provide a new insight into the pathological mechanisms of the white matter disease.

## Materials and Methods

### Subjects

We recruited 2185 subjects for this study. We excluded 390 subjects due to lack of DNA, or insufficient health-related and/or imaging data. Finally, a total of 1,795 subjects (1,002 males, 793 females) with a mean age of 60.1 ± 8.9 years (range 29 to 95) were included in this study. All subjects were voluntarily participated in a brain health screening program at Shimane Health Science Center between October 2001 and July 2013. The screening program included general medical and neurological examinations, MRI scans of head and blood tests. This study was in accordance with the Code of Ethics of the World Medical Association (Declaration of Helsinki), and was approved by the Ethical Committee of Shimane University School of Medicine. All participants have given informed written consent to use the data for this study. Personal and health-related history including socio-economic status, duration of school education, smoking and drinking habits, and medical history including hypertension, diabetes and dyslipidemia were obtained using questionnaire. There was no drug history of steroid use. Hypertension was defined as the blood pressure level at or above of 140/90 mmHg, or the use of antihypertensive drugs. Diabetes was defined as fasting blood glucose levels above 126 mg/dL, or the use of antidiabetic drugs. Hyperlipidemia was defined as the serum cholesterol levels above 220 mg/dL, or the use of lipid lowering drugs. Hemoglobin A1c (HbA1c) and blood glucose levels were evaluated using JDS (Japan Diabetes Society) references. To determine NGSP values, which is the international standard value of HbA1c, calculation was done using the following conversion equation: HbA1c (NGSP) = 1.02 × HbA1c (JDS) + 0.25^44^. The reference value of blood glucose is 73∼109 mg/dL, and that of HbA1c (JDS) is 4.3%∼5.8% in Japan. The reference value for plasma CST3 in Japanese population aged 51 to 75 years is 0.64 to 1.05 mg/L^45^.

In blood samples, total serum cholesterol, high density lipoprotein cholesterol, fasting blood glucose, HbA1c and CST3 were measured. Total serum cholesterol, high density lipoprotein cholesterol, fasting blood glucose, and HbA1c were measured using an automated biochemical analyzer at Shimane Health Science Center. CST3 was measured using a high sensitive gold colloid aggregation immunoassay kit (Alfresa Pharma Co., Osaka, Japan) and an automatic biochemical analyzer (BM6070; JEOL Ltd., Tokyo, Japan) at Central Clinical Laboratory division of Shimane University Hospital. Estimated glomerular filtration rate (eGFR) was calculated using a creatinine-based formula recommended by the Japanese Society of Nephrology^46^.

### Cognitive tests

Cognitive tests were done using Okabe’s Intelligence Scale, Kohs Block Design Test, Self-rating Depression Scale (SDS), Apathy Scale and Frontal Assessment Battery (FAB) tests. Okabe’s Intelligence Scale is a simple revision of Wechsler adult intelligence test, which is an examination that distinguishes and demarcates the difference between normal aging related mental changes, and mild to moderate dementia. Kohs Block Design Test is used in the field of neuropsychology as a screening test for mild cognitive impairment. SDS is a self-assessment scale consisting of 20 questions, which evaluates depression status of the subject. Apathy was assessed using Japanese version of apathy scale. This scale was used in a self-assessment style. The FAB was developed to screen functional elements of the frontal lobe multilaterally. The scores of all of the tests were evaluated blindly.

### MRI

The presence or absence of cerebral white matter lesions including periventricular hyperintensity (PVH) and deep and subcortical white matter hyperintensity (DSWMH) were evaluated by T2-weighted MRI. Both PVH and DSWMH were evaluated according to the Fazekas rating scale^47^. PVH was classified as grade 0: absence, grade 1: “cap” or pencil-thin lining, grade 2: Smooth “halo”, grade 3: irregular PVH extending into the deep white matter. We considered subjects of grade 0 as PVH free, and that of 1–3 as positive PVH. DSWMH was classified as grade 0: absent, grade 1: punctate foci, grade 2: beginning confluence foci and grade 3: large confluent areas. Grade 0 was considered as DSWMH free, and grade 1–3 as positive DSWMH. Both PVH and DSWMH were divided into negative and positive to assess the association from the early stages of white matter lesions.

### Genotyping

Based on previously published reports, we have analyzed 7 SNPs of *CST3* including −82G/C (rs5030707), −78T/G (rs71334202), −5G/A (rs113065546), +4A/C (rs73318135), +87C/T (rs1055084), +148G/A (rs1064039) and +213G/A (rs201089355). The genotyping was done with a TaqMan genotyping assay kit, using 7900 HT Fast Real Time PCR system (Thermo Fisher Scientific, USA). Since −78T/G is adjacent to −82G/C, a haplotype analysis was carried out by sequencing the area of 100 subjects. It was confirmed that −78 T/G and −82G/C are haplotype. Sequence analysis was performed using an ABI PRISM 310 Genetic Analyzer system (Thermo Fisher Scientific, USA).

### Statistics

Data was expressed as mean ± SD. Group comparisons were performed using Student *t* test for parametric variables and Mann-Whitney U test for nonparametric variables. Comparisons among the 3 *CST3* polymorphism groups were done using Kruskal-Wallis test. To investigate the association between PVH, DWMH and *CST3* gene polymorphism, logistic regression analysis was performed. In logistic regression analysis, PVH and DWMH were divided into two groups, and the independent variables were chosen considering multicollinearity. Age, gender, BMI, hypertension history, diabetes history, hyperlipidemia history, smoking history, drinking history, years of education, eGFR, and *CST3* gene polymorphism were analyzed as independent variables. All statistical analysis was performed using IBM SPSS Statistic software (SPSS Statistics 22). Statistical significance was defined as *p* < 0.05.
